# Current use and future potential of (physiologically based) pharmacokinetic modelling of radiopharmaceuticals: a review

**DOI:** 10.7150/thno.77279

**Published:** 2022-11-14

**Authors:** Hinke Siebinga, Berlinda J. de Wit-van der Veen, Marcel D.M. Stokkel, Alwin D.R. Huitema, Jeroen J.M.A. Hendrikx

**Affiliations:** 1 Department of Pharmacy & Pharmacology, The Netherlands Cancer Institute, Amsterdam, The Netherlands.; 2 Department of Nuclear Medicine, The Netherlands Cancer Institute, Amsterdam, The Netherlands.; 3 Department of Clinical Pharmacy, University Medical Center Utrecht, Utrecht University, Utrecht, The Netherlands.; 4 Department of Pharmacology, Princess Máxima Center for Pediatric Oncology, Utrecht, The Netherlands.

**Keywords:** population pharmacokinetic model, PBPK model, pharmacokinetics, PK model, radiopharmaceuticals

## Abstract

**Rationale:** Physiologically based pharmacokinetic (PBPK) and population pharmacokinetic (PK) modelling approaches are widely accepted in non-radiopharmaceutical drug development and research, while there is no major role for these approaches in radiopharmaceutical development yet. In this review, a literature search was performed to specify different research purposes and questions that have previously been answered using both PBPK and population PK modelling for radiopharmaceuticals.

**Methods:** The literature search was performed using the databases PubMed and Embase. Wide search terms included radiopharmaceutical, tracer, radioactivity, physiologically based pharmacokinetic model, PBPK, population pharmacokinetic model and nonlinear mixed-effects model.

**Results:** Eight articles and twenty articles were included for this review based on this literature search for population PK modelling and PBPK modelling, respectively. Included population PK analyses showed to have an added value to develop predictive models for a population and to describe individual variability sources. Main purposes of PBPK models appeared related to optimizing treatment (planning), or more specifically: to find the optimal combination of peptide amount and radioactivity, to optimize treatment planning by reducing the number of measurements, to individualize treatment, to get insights in differences between pre-therapeutic and therapeutic scans or to understand inter-patient differences. Other main research subjects were regarding radiopharmaceutical comparisons, selecting ligands based on their peptide characteristics and gaining a better understanding of drug-drug interactions.

**Conclusions:** The use of PK modelling approaches in radiopharmaceutical research remains scarce, but can be expanded to obtain a better understanding of PK and whole-body distribution of radiopharmaceuticals in general. PK modelling of radiopharmaceuticals has great potential for the nearby future and could contribute to the evolving research of radiopharmaceuticals.

## Introduction

Clinical applications of radioactive pharmaceuticals (radiopharmaceuticals) already originate from the 1920s, but their use is growing and currently radiopharmaceuticals are considered highly valuable agents for diagnosis and treatment of several diseases 1. The increasing interest in (theranostic) radiopharmaceuticals was also acknowledged by the United States National Cancer Institute, since they launched the Radiopharmaceutical Development Initiative (RDI) in 2019. This RDI had the objective to promote promising new radiopharmaceuticals into clinical trials and integrate pharmacology, cancer biology and dosimetry into these trials 2. Towards clinical application, radiopharmaceuticals (and radiopharmaceutical kits) are eventually mandated for registration by the regulatory agencies (the United States Food and Drug Administration (FDA) and the European Medicines Agency (EMA)) 3. For this approval, all pharmacologic, toxicological and animal testing is required for all radiopharmaceuticals (with exceptions for some microdose trials 4), as they are treated as 'normal' drugs.

In recent years, drug development and approval was increasingly supported by model-informed drug development (MIDD), which was also encouraged by the FDA in their Critical Path Initiative (CPI) and the Model-Informed Drug Development (MIDD) Pilot Program 5, 6. MIDD approaches help to accelerate drug development and regulatory approval, by using quantitative methods to inform decisions, such as clinical trial design, efficacy and safety evaluation (e.g. by predicting target and organ exposure), selecting favorable compounds, finding optimal dosing regimens or by gaining knowledge regarding potential drug-drug interactions (DDIs) or receptor saturation statuses in case of receptor-targeted therapy. Two important approaches that are commonly used for MIDD are physiologically based pharmacokinetic (PBPK) models and population pharmacokinetic (PK) models. PBPK models help to gain more insights in PK behavior, by combining drug-specific information with physiological system-specific parameters in a multi-compartment model to predict whole-body distribution. Another, more general, modelling approach is using population PK models, where several central or peripheral compartments describe PK behavior for the population of interest and the PK variability within this population.

Despite the fact that these PBPK and population PK modelling approaches are widely accepted in non-radiopharmaceutical drug development and research, and although requirements for approval of radiopharmaceuticals are similar to 'normal drugs', there is no major role for MIDD in radiopharmaceutical development yet 7, 8. Since these modelling approaches are rather new concepts for radiopharmaceuticals, more information is needed to further explain the potential of both these PK modelling methods for nuclear medicine research. Especially, since these PK modelling approaches will help to improve drug-development in various stages of research prior to approval, but also could enhance the evaluation and optimization of radiopharmaceutical use in clinical practice. Hence, this review will provide an overview on the applications of these modelling approaches. Second, to get a detailed insight in the added value of PK modelling for radiopharmaceuticals, a literature search was performed to specify different research purposes and questions that have previously been answered using both PBPK and population PK modelling for radiopharmaceuticals. Lastly, future perspectives of PK modelling will be further explicated.

### Population PK modelling

Population PK modelling is the study to obtain a model that describes concentration-time profiles at a population level based on dose input information, with the additional aim of trying to identify PK parameters and (sources of) variability in the population of interest 9, 10. Although several different approaches for PK analysis exist (e.g., non-compartmental, naïve pooled and two-stage analysis), population PK studies are most frequently performed by nonlinear mixed-effects modelling (NLMEM) (see Supplementary Materials for additional information regarding NLMEMs). Compartmental population PK models typically consist of a central compartment, which can be linked to several additional peripheral compartments via rate constants 11. The model output parameters contribute to gaining better understanding of clearance (CL) and volume of distribution (Vd) of the drug of interest. In the end, the goal is to describe PK behavior in selected compartments and not to describe full physiological behavior. To develop such population PK models retrospective data are used (e.g. (blood) samples previously derived from clinical studies or routine clinical care), but there is no need for many observations per individual or structured sampling time schedules 10. Instead, it is an advantage that few observations (sparse data) from many individuals can be used to develop population PK models and structured prospective sample protocols are not required. Still, using population PK models, individual PK parameters can be estimated with Bayesian estimation also for patients where extensive PK sampling was not feasible. Furthermore, these models could relate PK of a drug to its clinical effect, also known as pharmacodynamics (PD). PK/PD models could include a relationship between PK and clinical outcome parameters, such as response rates, tumor growth or biomarkers. On the other hand, toxicity can also be related to PK, for example based on Common Toxicity Criteria grades. A more detailed description about development and evaluation of population PK models can be found in selected key publications 9, 10, 12, 13.

In general, population PK models are useful to obtain information about population PK behavior and its variability within the population of interest. In addition, these analyses help to identify relevant factors (covariates) that can affect drug exposure, such as body weight, renal function or hematological values. Population PK models are regularly applied in drug development, but can also be used in direct patient care. In this latter case, the models are useful to, for example, design the best possible individualized initial dosage regimen and optimize treatment based on estimated individual PK parameters and thus estimated exposure 11, 14. In addition, population PK modelling includes more advantages, such as explaining differences between subgroups in responses to drug exposure and they can play a major role in the development and evaluation of dosing strategies. These models are regularly developed to select safe and effective drug dosing regimens. Hence, population PK modelling is often used in all different phases of drug development and registration 15. The FDA and EMA also provided guidelines to assist parties in the application of population PK modelling and provide guidance on how to report results from such analyses for regulatory procedures 8, 16.

### PBPK modelling

PBPK models are mathematical models that help to understand and predict the PK of drugs. A 'bottom-up' approach is used for PBPK model development, meaning that these models are based on the physical and chemical properties of a drug (drug-specific knowledge) and the independent prior knowledge on the physiology and biology at the organism level (system-specific knowledge) 17, 18. This is different compared to population PK models, which use a 'top-down' concept where observed PK data forms the basis for model development 19. Another difference compared to typical population PK models is that PBPK model structures are more complex, since they consist of multiple compartments representing the total physiology of the organism, where in population PK models only a limited number of lumped compartments is used to describe the data.

PBPK models include different building blocks with information to generate predictions. These building blocks are divided in organism, drug, study protocol and formulation properties 17. The physiological part of whole-body PBPK models contain an explicit representation of all organs and tissues that have a relevant impact on the absorption, distribution, metabolism and excretion of the drug*.* The organs are linked by arterial and venous blood compartments. In addition, each organ is further characterized by parameters such as a specific blood flow, volume, tissue-partition coefficient and permeability 17, 20. Such system-specific information is often based on reference population values, but can also be informed based on (measured) clinical parameters derived from specific subpopulations. Since all these different compartments are included, the model is a comprehensive structural representation of the physiology of an organism. Drug-specific parameters can be estimated from physical and chemical properties or can be measured *in vitro* or *in vivo* 17. Drug-specific input parameters that are obtained by *in vitro* studies are used for instance to predict plasma and tissue concentration-time profiles by the *in vitro-in vivo* extrapolation (IVIVE) technique 11. Combining the information from these building blocks will eventually lead to a mechanistic representation of the drug in biological systems, so that drug concentration-time profiles can be predicted *a priori* 17, 20. Predictions can either be individual or population based, where in the latter case population variability in input parameters is taken into account. Furthermore, it is possible to obtain concentration-time profiles within different compartments, leading to a detailed whole-body distribution profile prediction. The PBPK model prediction output can be evaluated based on clinical data, so that model predictions describe the clinical observed data best. However, clinical data collection is not necessarily needed, and one could also predict a PK profile for a new chemical compound or a known compound in another species based on physiology and physicochemical properties only 18. Of course, such a model would contain many assumptions and evaluation of predicted concentration-time profiles is not possible.

PBPK models are useful to understand (whole-body) PK, select the most promising structure analogue for further development and to extrapolate findings to different species, populations or disease states. In addition, an advantage of these models is that the output PK parameters are more related to the species' physiology and drug properties compared to PK output in typical population PK models. A brief overview on PBPK models was provided above and any further information regarding PBPK modelling can be found in literature 11, 17-21.

A growing number of regulatory submissions of non-radioactive drugs include PBPK models and, therefore, the EMA and FDA provided guidelines on the reporting of PBPK modelling and simulation to give a detailed advice on what to include in a PBPK modelling report 7, 22. PBPK modelling is used in regulatory submissions for two main purposes, namely to qualitatively and quantitatively predict DDIs and to support or predict initial dose selection in paediatric or other specific patient groups and first-in-human trials. PBPK models are a very useful tool for extrapolations outside the studied scenarios or studied populations. Zhuang *et al*. provided a clear overview of cases where PBPK modelling was used in drug development research 23. Subjects of these cases were candidate drug evaluation, DDI prediction, human PK and DDI prediction to avoid clinical DDI trials, dose guidance for renal impairment and bridge healthy adults to special populations. Besides, it was shown that in less than six years (from 1^st^ July 2008 to 31^th^ December 2013) 112 PBPK packages were submitted to the FDA, of which most were DDI related 23.

## Methods

The literature search was performed in September 2022, using the databases PubMed and Embase. Wide search terms included radiopharmaceutical, tracer, radioactivity, physiologically based pharmacokinetic model, PBPK, population pharmacokinetic model and nonlinear mixed-effects model. Detailed information on the search terms is provided in the Supplementary Materials. The initial screening consisted of title evaluation to ascertain their relevance. Afterwards, abstract and/or full texts of the selected papers were evaluated. Reference lists of selected manuscripts were verified to identify relevant additional literature. Results were limited to publications in English, original research papers and models for human applications only. Articles were included in this review based on the following requirements: a) a radiopharmaceutical was the drug of interest, and b) the presence of a PBPK or population PK(PD) model to assess PK of the specific radiopharmaceutical. To clarify, only studies that were focused to assess PK of the specific radiopharmaceutical were included, so models where medical isotopes were used to evaluate renal function or receptor status were not included. In addition, microtracer studies, that were performed to obtain plasma concentration of non-radioactive drugs, were also excluded from analysis. For population PK models, this review focused on NLMEMs only. From all included articles, research aims and purposes were extracted and categorized to different main topics.

## Results

**Figure 1:**
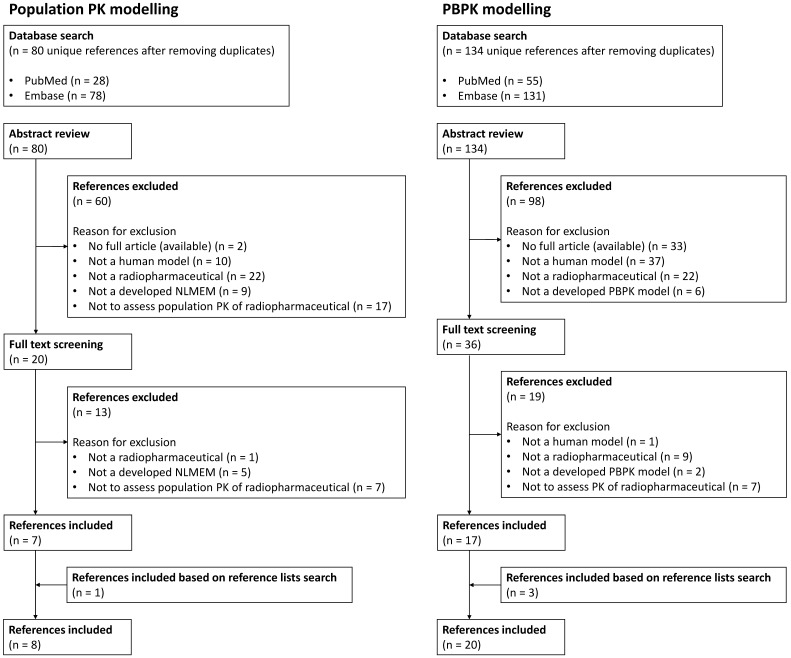
Overview of literature review selection process for both population PK and PBPK modelling results.

The primary literature search identified 214 articles, 80 regarding population PK modelling and 134 regarding PBPK modelling. After screening and removing duplicates, seven and seventeen articles were included for this review based on the literature search for population PK modelling and PBPK modelling, respectively. Besides, one and three additional articles regarding population PK modelling and PBPK modelling, respectively, were included based on search in reference lists. An overview of the reference selection process is provided in Figure 1. Overviews of the included references are shown in Table 1 and Table 2, representing literature for population PK and PBPK modelling, respectively.

### Current application of population PK modelling in radiopharmaceutical research

Few articles have been published describing population PK models for radiopharmaceuticals. In general, population PK models provide insights in population PK parameters and their variability within a population. A clear example of such an approach was given for the use of radioiodine (Iodine-131 sodium iodide ([^131^I]I-NaI)) in thyroid cancer patients after thyroidectomy 24. Based on blood sample and SPECT scan data, a multi-compartment model was developed to describe PK for radioiodine in this specific patient group. Model predictions were in good agreement with observed whole-body, thyroid (remnant), blood and protein bound iodine concentrations. Model results of this specific population showed a lower uptake rate constant for thyroid and a lower distribution rate from blood into different compartments, although urinary excretion was faster compared to a model based on healthy patient data (including different uptake scenarios). Unfortunately, no covariates were included in this model, thus inter-individual random effects remained unexplained. This work showed the need to implement population specific rate constants within the commonly used ICRP-128 iodine model (International Commission on Radiological Protection), instead of assuming normal iodine PK for the entire human population. This modification allows for more precise assessments of radiation protection requirements for thyroid cancer patients receiving radioiodine.

In addition to describing population PK parameters, population PK models are often used to assess the effect of covariates on these PK parameters. Covariates can be defined as patient characteristics or demographics, but also other factors such as the addition of co-therapy can be assessed as a potential covariate on PK of a drug. This latter approach was used by Puszkiel *et al.*, where a three-compartment model was developed to evaluate the interaction of amino acid co-infusion on Lutetium-177 (^177^Lu) DOTATATE PK 25. The co-infusion of amino acids seemed to have a significant effect on [^177^Lu]Lu-DOTATATE PK, namely an increase in the elimination rate constant from 0.204 to 0.306 h^-1^. However, this covariate effect was associated with a high inter-individual variability (104%). This also contributed to a high inter-individual variability in hematotoxicity, since an increased plasma exposure was associated with decreased lymphocyte count. Lambert *et al.* also assessed the covariate effect of amino acid co-infusion on [^177^Lu]Lu-DOTATATE PK, although in this study two amino acid solutions (Primene^®^ and Lysakare^®^) were compared 26. Again, both amino acid co-infusions showed to have a significant effect on the elimination rate constant. However, Primene^®^ significantly increased the elimination rate constant by a 1.73-fold, while Lysakare^®^ co-infusion decreased the [^177^Lu]Lu-DOTATATE elimination rate constant by a 1.67-fold. Results also indicated a trend towards higher toxicity with Lysakare^®^, namely a greater reduction in lymphocyte count. These two population PK models are good examples of using radioactivity-time data to identify covariates on [^177^Lu]Lu-DOTATATE PK within a population.

Identification of the impact of patients' demographic and clinical characteristics as covariates on PK was also performed for other radiopharmaceuticals 27, 28. Topić Vučenović *et al.* developed a two-compartment model (representing blood pool and thyroid tissue) for radioiodine therapy based on thyroid uptake data from 345 patients with benign thyroid disease 27. Patient characteristics that were tested as covariates were age, gender, clinical diagnosis and functional thyroid volume. In addition, several parameters describing thyroid function were evaluated as covariates, namely thyroid-stimulating hormone, free thyroxine, previous therapy with anti-thyroid drugs and time of therapy discontinuation before uptake measurements. Covariate testing results showed that clinical diagnosis, age, functional thyroid volume, free thyroxine in plasma, use of anti-thyroid drugs and time of discontinuation of therapy before administration of radioiodine had a significant impact on uptake into the thyroid tissue. In addition, age had a significant effect on the effective half-life of radioiodine. Inclusion of these covariates into the developed model described, and thus decreased, the inter-individual variability on uptake into thyroid tissue. These covariate modelling results could be used to also explore potential correlation between (predicted) thyroid uptake and outcome of therapy. Moreover, therapy might be individualized based on these results, since the final model can estimate individual radioiodine PK parameters based on demographic patient data and clinical characteristics.

Another example for covariate modelling is a model developed by Van Rij *et al.* to identify patient characteristics that contribute to alterations in PK of Carbon-11 (^11^C) flumazenil 28. Their research aims were to develop a population PK model, to identify patient characteristics that influence PK of [^11^C]C-flumazenil and to use the model to define and validate an optimal sampling protocol for PET studies. A two-compartment model described the data best, with significant covariates being type of disease for CL and weight for central Vd. To explicate, patients with epilepsy showed a decrease in CL of 20% and the influence of body weight on Vd was an increase of 0.55% per kg scaled to a mean weight of 70 kg. Using the estimated PK parameter results, an additional simulation analysis resulted in optimized sampling times at 30 and 60 min.

Population PK models can also be useful to optimize measurement time points, which in case of radiopharmaceutical research is often applied to reduce the number of imaging time points needed to determine accurate individual time-activity curves or absorbed doses 29-31. Merrill *et al.* and Melgar Pérez *et al.* focused on optimizing individual absorbed doses after radioiodine therapy (^131^I and Iodine-123 (^123^I)) in patients with Graves' disease. Merrill *et al.* assessed the optimal sampling times for different target variables: time of maximum activity, maximum fractional thyroid uptake, time-integrated activity and effective half-life 29. For time-integrated activity and effective half-life the optimal 1-point sampling time was as late as possible (around 1 week). For accurate estimation of maximum uptake and time of maximum activity, two measurements were required and a third measurement resulted in a small additional improvement. Going from one to two measurements resulted in the best gain in accuracy for all target variables. Melgar Pérez *et al.* also quantified the accuracy of different time-sampling sequences and showed that the last measurement at 96 h was the most important for radioiodine therapy 30. Also, comparable to results by Merrill *et al.*, a third measurement provided only a small improvement and thus their 3-point schedule (4, 24 and 96 h) was adjusted to a 2-pointed schedule (4 and 96 h). A similar approach was performed by Devasia *et al.* for [^177^Lu]Lu-DOTATATE in patients with neuroendocrine tumors (NETs) 31. NLMEMs were fit using either 1 or 2 time points and subsequent calculated time-integrated activities were compared to values from other reduced-time-point methods. Results showed that NLMEMs resulted in lower bias, less variability and fewer outliers compared to other methods (mono- and bi-exponential models). This study also nicely demonstrated that NLMEMs can improve time-integrated activity estimations for individual kidney dosimetry.

The discussed articles regarding population PK models showed that these models can help to get insight in population PK parameters, but also in variability on these parameters within a population. Furthermore, these models play a role in identifying factors or patient characteristics that can explain variability in these PK parameters. These approaches could eventually lead to optimization of therapy, for example by individualizing dosing based on these covariates resulting in a more personalized medicine approach or by investigating optimal sample or scan time points and thus reducing patients' burden.

### Current application of PBPK modelling in radiopharmaceutical research

A total of twenty published PBPK models regarding different radiopharmaceuticals were evaluated to gain insights in different research purposes and knowledge gaps that can be answered using PBPK modelling. This might help highlighting different opportunities and the added value of PBPK modelling for radiopharmaceutical research in general.

Our group has developed a PBPK model to assess organ distribution of Gallium-68 (^68^Ga) DOTATATE in patients without NETs 32. By eliminating distribution to tumors that may affect organ uptake, all other relevant parameters were identified more accurately. Results showed that variability in SSTR expression as well as different administered peptide amounts had a major impact on tissue distribution. This study is a straightforward example of describing whole-body distribution using a PBPK model. Another, rather simplistic, approach of PBPK modelling was performed by Gospavic *et al.* 33, with a remarkably general objective to assess the benefits of PBPK models in patients with NETs treated with [^177^Lu]Lu-DOTATATE. The blood flow restricted (or perfusion rate limited) model consisted of five organ compartments (lungs, kidney, liver, brain and rest) and a venous and arterial blood compartment. Only whole body planar scintigraphy data were used to evaluate the model and the authors concluded that this was sufficient for prediction of the biodistribution and absorbed doses in this small patient group 33. The predicted time-activity curves were only evaluated by visual inspection of predicted versus measured values. Both accuracy of tissue accumulation profiles particularly in the abdomen and the goodness-of-fit evaluation are debatable.

This prediction of biodistribution of a radiopharmaceutical represents a main achievement of PBPK modelling. However, in many previously published models this approach was extended and authors tend to improve radiopharmaceutical treatment using different concepts. One manner to optimize treatment is to investigate the optimal combination of administered peptide amount and radioactivity 34-38. Kletting *et al.* studied the effect of peptide amount and activity for peptide receptor radionuclide therapy (PRRT) with Yttrium-90 (^90^Y) DOTATATE to estimate a given maximal kidney biological effective dose (BED) 38. This strategy is useful for optimizing PRRT treatment planning for NETs based on a pre-defined maximal radiation dose to organs at risk (OARs). A whole-body PBPK model showed good fits after evaluation with clinical data (based on Indium-111 (^111^In) DOTATATE imaging) and comparing values of estimated parameters with literature values. Simulations for optimal kidney BEDs were performed, resulting in suggestions for individualized optimal peptide amount and activity combinations 38. A similar approach was performed by Jiménez-Franco *et al.* 36, to optimize the treatment planning in patients with NETs or meningiomas. Again, the maximum BEDs of OARs were considered, but also effects of multiple tumor lesions and a maximum achievable molar activity on the total number of killed tumor cells were evaluated and therewith strategies for optimizing tumor control compared to the 'typical' PRRT plan. However, it should be noted that, since this was an *in silico* study, all estimations regarding cell death were based on fixed assumptions as well as defined BED limits for OARs, which is challenging since such assumptions are often derived from external radiation therapy 39. Extrapolation of knowledge regarding cellular effects from external radiation therapy to radionuclide therapy is not straightforward as both radiation delivery profiles and linear energy transfer differ greatly.

A similar concept of considering maximum BEDs to tumors and organs was used by Begum *et al.* for patients receiving radioligand therapy with ^177^Lu-prostate-specific membrane antigen (PSMA) for metastatic castration-resistant prostate cancer 35. Their aim was to predict the effect of total tumor volume and different peptide amounts on maximum BEDs. Tumor volumes ranged from 0.1-10 L and tumor growth was explicitly modelled. Peptide amounts varied from 2-2^10^ nmol. The predictions showed that an increase of tumor volume resulted in a decrease in BEDs to the tumor lesions and organs (except for bone marrow). This resulted in an approach where patients with large PSMA-positive tumor volumes (>300 mL) could receive a higher activity (10.4 ± 4.4 GBq) and peptide amount (273 ± 136 nmol) to maximize the BED in tumors and not exceed the tolerable BED in organs 35. This research group also investigated the effect of peptide amount, affinity and internalization in PSMA imaging and therapy 34. The previously developed PSMA PBPK model, based on data from imaging with PSMA-11, was reused to investigate the interconnected effect of affinity, internalization and administered ligand dose. Normalized activity concentrations were predicted for tumor lesions, background and several organs (at risk) for both imaging and therapy. For imaging, predictions indicated a maximal improvement in tumors, regarding normalized activity concentrations, with a PSMA ligand amount of 32 nmol (obtained with a dissociation constant (K_D_) of 0.01 nM). Internalization rate did not substantially affect normalized activity concentrations. For therapy, the highest absorbed dose was also achieved after administration of 32 nmol. Higher peptide amounts resulted in a decrease in tumor absorbed doses, especially in highly perfused tissue for high affinities. The internalization rate variation resulted in differences in absorbed doses, depending on the K_D_ and ligand amount. Optimal combinations of internalization rate, association (k_on_) and dissociation (k_off_) rate constants and ligand amount were investigated. Using these predictions, therapy might be improved by choosing optimal activity and ligand amounts (i.e. specific activity), so that highest tumor-to-background ratios will be achieved 34.

Another way to apply PBPK modelling to optimize treatment is by predicting time-integrated activity coefficients (TIACs) or optimizing this prediction. Such approaches were published by Hardiansyah *et al.* 40-42. These studies all contribute to improve the prediction of biodistribution based on prior knowledge, for example by prediction of TIACs (and investigated the accuracy) using simulated PET measurements before PRRT using PBPK modelling 40. A previously published PBPK model was used 43 to model distribution of [^90^Y]Y-DOTATOC in metastasized NET patients. Results showed a good fit of the model to the biokinetic data, based on visual inspection, an adjusted R^2^ of ≥0.96 and coefficients of variation of the fitted parameters <0.3. Predicted organ (liver, spleen and kidney) TIACs were accurate, although individual and population tumor variabilities were not (relative variability values >15%, while ≤10% was assumed accurate). Still, it was concluded that using only two measurements for [^68^Ga]Ga-DOTATATE PET (at 1 and 4 h post injection) therapeutic biodistribution can be estimated with acceptable accuracy. However, this only applied to the (small) studied patient group and the tumor volume has to be known exactly 42. Nevertheless, these results might lead to clinical therapy improvements achieved by a lower number of scans needed, which of course is in favor of both patients and clinicians.

In line with this treatment planning approach, investigating the effect of a reduced number of measurement points on treatment planning using PBPK models was also introduced by Rinscheid *et al.* to improve accuracy and precision of [^111^In]In-DOTATATE dosimetry 44. One major finding was the identification of the importance of one late scan time point (at day 5 or 6 post injection). Maaβ *et al.* also developed such a PBPK model for PRRT using [^111^In]In-DTPAOC (Octreoscan^®^) 45. Using pre-therapeutic data of only 15 NET patients, TIACs were calculated for tumors and several other compartments. It was concluded that the absorbed dose in kidneys could be determined with acceptable accuracy using only two time points (4 h and 2 days post injection) 45. Thus, in both these papers, PBPK modelling was used to reduce intensive data needed for individual dosimetry and therefore optimizing treatment planning.

Besides optimizing scan time measurements, PBPK modelling may be used to individualize treatment planning, which was already demonstrated for ^90^Y-labeled anti-CD66 radioimmunotherapy 46 and PRRT using [^90^Y]Y-DOTATOC 47. In addition, Kletting *et al.* used a PBPK model to individually predict tumor response after radioligand therapy (based on pre-therapeutic imaging) using [^177^Lu]Lu-PSMA I&T in metastatic castration resistant prostate cancer patients 48. An exponential growth model was used to describe tumor growth, while for tumor reduction a linear quadratic model was included. Evaluation of this approach was performed by comparing predicted and measured tumor volume for 13 metastatic castration resistant prostate cancer patients 6 weeks after therapy and this evaluation showed a small mean (±SD) relative deviation of 1% (± 40%) (excluding 1 patient).

In line with personalized treatment planning or response predictions, PBPK models might help to gain insights in intra-patient differences between pre-therapeutic and therapeutic scans. This was shown in a previously described paper 46, but has also been investigated for [^90^Y]Y-DOTATATE, where a PBPK model was developed for PRRT to investigate the differences in predicted and actually absorbed doses 43. Predictions were made for various amounts of [^90^Y]Y-DOTATATE (10-200 µg). One of their major results was that different peptide amounts resulted in large differences of pre-therapeutic and therapeutic TIACs (residence times) for all somatostatin receptor (SSTR) 2 positive organs, although no optimal peptide amount was recommended based on the results 43.

One last approach to optimize treatment for each individual patient, is using PBPK models to better understand inter-patient variability in radiopharmaceutical distribution. The inter-patient variability for absorbed doses to kidneys and tumor lesions after [^177^Lu]Lu-PSMA therapy was recently studied, showing high variability in absorbed doses to kidneys and tumors (coefficient of variation 31-59%) in the observed population 49. Results of the PBPK model showed that tumor receptor density and release rates were two main parameters to determine inter-individual variability in absorbed doses to tumor lesions. For kidney, age-independent kidney flow, receptor density and kidneys release rate were identified as the most important factors to affect inter-patient variability. Therefore, the authors suggest that treatment planning might be individualized by determining individual values for the previously mentioned parameters before start of therapy.

Indirectly related to individual improvement of radioligand therapy based on tumor physiology, tumor physiology information can be used to select specific ligands or even patients for therapy 50. Jiménez-Franco *et al.* managed to determine a minimal tumor perfusion and receptor density for an optimal tumor control probability in the treatment of NETs and meningioma using the standard treatment with [^177^Lu]Lu-DOTATATE. In addition, other treatment strategies were examined based on tumor control probability and maximum tolerated BEDs for OARs. Results showed highest tumor control probability, without exceeding maximum BEDs for OARs, for the strategy based on estimated optimal ligand amount and activity per patient. Interestingly, simulations indicated that patients with limited tumor receptor density and/or tumor perfusion might not benefit from [^177^Lu]Lu-DOTATATE treatment using the standard therapy protocol. Therefore, this PBPK model could be of importance in patient selection for therapy. In addition, the authors mention that this method could also be used in radiopharmaceutical development, to select ligands for specific types of tumors (with specific tumor physiologies) to achieve the highest response rate.

PBPK modelling can also play an important role in drug development and regulatory submissions, as was already pointed out. A mechanistic modelling approach to predict or confirm and thus also understand potential DDIs will help to simplify DDI research in both drug development, radiopharmaceutical selection and regulatory submission. Our literature search resulted in only one publication regarding DDIs for radiopharmaceuticals 51. In this paper the effect of potential inhibition of the biliary multidrug resistance-associated protein 2 transporter by ritonavir on Technetium-99m (^99m^Tc) mebrofenin (hepatic) exposure was investigated using a semi-PBPK modelling approach.^ [99m^Tc]Tc-mebrofenin is an imaging agent that is used to diagnose disorders of the hepatobiliary network and gallbladder. A sensitivity analysis of the PBPK model indicated that not one single, but a combination of factors was necessary to describe the effect of ritonavir leading to an increased observed [^99m^Tc]Tc-mebrofenin blood exposure. In addition, both clinical data and PK analysis results showed that ritonavir did not significantly affect [^99m^Tc]Tc-mebrofenin biliary CL. This paper presented an excellent example of the combination of *in vitro* and *in silico* research approaches to predict *in vivo* effects.

Lastly, PBPK models could provide information regarding comparing different radiopharmaceuticals. This was recently performed by Bartelink *et al.*, where three epidermal growth factor receptor (EGFR) tyrosine kinase inhibitors (TKIs) were compared, namely [^11^C]C-erlotinib, Fluorine-18 (^18^F) afatinib and [^11^C]C-osimertinib 52. The developed mechanistic model included system-specific information regarding non-small cell lung cancer key hallmarks, as well as different physicochemical and drug-specific properties for all three compounds. Comparison of the three EGFR-TKIs was based on whole-body distribution and their target uptake by predicting the tumor-to-lung ratios. Model predictions showed extensive distribution to most tissues for osimertinib and afatinib, while for erlotinib low tissue distribution and high whole blood concentrations were predicted. In addition, tumor-to-lung contrast was predicted (within established boundaries) >1 for erlotinib and afatinib, while for osimertinib this was <1. This article provided an evident comparison of three different radiopharmaceuticals based on tissue distribution, which represent clinically relevant endpoints.

## Future perspectives and challenges for the application of PK modelling to radiopharmaceuticals

**Figure 2:**
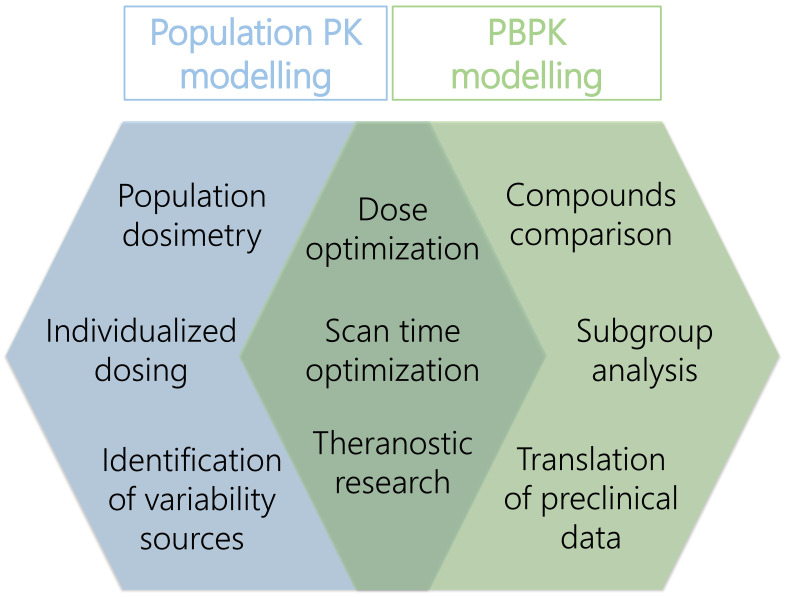
Overview of main subjects of PBPK and population PK modelling approaches that could contribute to improve radiopharmaceutical research.

The interest in PK modelling of radiopharmaceuticals has increased over the last years. This review provided an overview of applications of population PK and PBPK modelling specifically focussed on radiopharmaceuticals and illustrated a variety of research questions that can be answered using these approaches. However, it became apparent that the total result of publications regarding (PB)PK models was rather limited, so there still are research challenges for PK modelling to be investigated. Here we will focus on additional benefits of population PK and PBPK modelling that may improve future research. Figure 2 shows an overview of the main benefits of both approaches for future radiopharmaceutical research.

### Population PK modelling

Compared to PBPK modelling, population PK modelling is not extensively used for radiopharmaceuticals yet. However, population PK analyses could have an added value to develop predictive models for a population and to describe individual variability sources, such as patient characteristics, clinical status, demographic factors or disease status. Although not extensive, this was investigated for some radiopharmaceuticals 24, 25, 27, 28.

#### Covariate modelling and optimizing (individualized) dosing

Insights in differences in PK parameters and its variability could help in the selection of appropriate dosing regimens and could even lead to a more personalized medicine approach. For this approach, population PK models are extensively used in drug development and optimization 11, 14, 15. In addition, an advantage of such models is that potential covariates that impact PK parameters can be addressed. This is a useful approach to assess factors that lead to altered distribution and might eventually even help to optimize dosing schemes. An example was already discussed in this review 25, where administration of the amino acid solution resulted in altered PK. This covariate modelling can be expanded, for example to evaluate whether octreotide or lanreotide dosing prior to PRRT significantly impacts PK, to assess the effect of aging on radiopharmaceutical distribution or to evaluate the effect of renal impairment on radiopharmaceutical CL. Identifying covariates on PK parameters can then result in, for example, adjusted dosing schedules for elderly or patients with renal impairment. These are just a few suggestions for the widespread application of covariate modelling using population PK models.

#### Population dosimetry

In our view, the use of population PK models can be specifically extended to radiopharmaceutical dosimetry. Currently, dosimetry of radionuclide therapies is mainly evaluated on an individual basis and insights in population dosimetry and distribution is not regularly obtained. Using absorbed dose information based on scans as an input for multi-compartment population PK models will result in population predictions for uptake in relevant organs or tumors (which have to be added as specific compartments). In addition, such an approach will give insights in inter-patient variability in organ and tumor uptake, and possibly even covariates describing parts of this variability will be revealed. In that case, population PK models could accomplish a personalized dosing approach, where individual uptake into different organs or tumors can be predicted based on patient characteristics. In addition, final population PK models can be used to optimize individual time-activity curve predictions based on limited post-treatment scans, to eventually also optimize absorbed dose calculations.

Also from a theranostic point of view, PK modelling approaches could improve dosimetry research. Though an important assumption in theranostics is that the diagnostic and therapeutic counterpart behave alike, retrospective studies showed varying degrees of concordance between pre-therapeutic and therapeutic accumulation of the radiopharmaceuticals 53-59. The discrepancies between pre-therapeutic and therapeutic biodistribution are attributed to factors like targeting peptide, peptide dosing, use of co-medication and/or chosen imaging time-points, which can be accounted for in these models. So, population PK models could provide information regarding (dis)similarities of both counterparts and bridge the gap between diagnosis and therapy evaluation by incorporating these conjectured factors. PBPK modelling can also play a role in such analyses, as was already discussed previously 43, 48. In addition, a recent position statement by the European Association of Nuclear Medicine (EANM) regarding the use of artificial intelligence identified implementation of PBPK modelling to enhance image interpretation as one of the challenges that need to be addressed in the nearby future 60. Applying PK modelling could eventually result in less invasive post-treatment scan protocols, optimized dosing based on diagnostic scans or even patient selection for therapy.

#### Optimizing scan protocols

Furthermore, population PK models are a useful tool to optimize sample or scan time protocols. Regarding radiopharmaceuticals, a population model could help to improve sampling protocols by reducing the amount of post-treatment scans required or by identifying most optimal times for those scans, which was already addressed by some articles 28-31. These approaches would really contribute to radiopharmaceuticals research in general, but more specifically also for PRRT or radioligand therapy since multiple post-treatment scans are currently required to evaluate therapy response. In such cases, only few observations will be needed to estimate full individual PK behavior based on a patient deviation from the population model. There are multiple examples of optimizing study or treatment designs with regards to measurement time points for conventional drugs 61-63.

#### Challenges

Of course, using population PK modelling approaches will also raise challenges that should be pointed out here. Firstly, those methods are mathematically and statistically complex and one has to specialize in the methodology to apply these models. Secondly, the developed model will be highly reliant on the quality of input data. Lastly, decisions made by the developer of the model during model optimization could also highly impact model results 9.

### PBPK modelling

As explicated in this review, PBPK models are a useful tool for many purposes. Data sources for PBPK models could be plasma concentrations, tissue concentrations or regional venous concentrations 18. Since tissue concentrations can be derived from scan data, radiopharmaceuticals are suitable components for PBPK modelling. Another general advantage of PBPK models is that they can be easily reused with rather simple adjustments. This causes that several research questions, even regarding different radiopharmaceuticals, can be answered using one structural (whole-body) PBPK model.

Next to these wide advantages of PBPK modelling for radiopharmaceuticals, there are many specific arguments in favour of promoting this concept for radiopharmaceuticals. For instance, as previously outlined, the main purposes of PBPK modelling of radiopharmaceuticals appeared related to optimizing treatment (planning). This goal can be further subcategorized in different specific subjects, namely to find the optimal combination of peptide amount and radioactivity, to optimize treatment planning by reducing the number of measurements, to individualize treatment (planning), to get insights in differences between pre-therapeutic and therapeutic scans and to understand inter-patient differences. Other main research subjects that were discussed in this review were: to select ligands based on their peptide characteristics, to gain a better understanding of DDIs and to directly compare radiopharmaceuticals.

#### Drug development and regulatory approval

As was previously specified, using PBPK models to predict and understand DDIs might be favourable in drug development and regulatory approval. However, in our view, PBPK models could play a broader role for these specific purposes. The EANM technologist's guide about radiopharmacy that was published in 2019 described a translational approach for radiopharmaceutical development 64. Several approaches were listed to explain how to provide necessary information for distribution and safety assessment, permitting characterization of potential adverse effects in humans. Although PBPK modelling was not described, it could definitely play an important part in these preclinical studies, since it can contribute to answer preclinical questions regarding PK profile predictions and toxicity in healthy tissues. *In vitro* studies can be used to predict *in vivo* distribution profiles in species, which is called *in vitro* to *in vivo* extrapolation (IVIVE). Although, this is ideally combined with the 'top-down' approach to optimize model parameters by taking advantage of observed (pre)clinical data 65. In the same way, preclinical animal data can be used to predict distribution and PK in humans 17. This could then result in fewer trials needed for determining PK in humans, finding initial dosing regimens and predicting uptake in healthy tissue leading to toxicity. In general, PBPK models are an efficient tool to speed-up the process prior to first human phase 1 trials 64.

#### Dose optimization

Furthermore, PBPK approaches are a powerful framework to examine differences between the amounts of peptides that are administered. Advantages regarding identifying an optimal dose regimen can specifically be of added value for radiopharmaceuticals, since there often is a large difference between peptide amounts for pre-therapeutic measurements and therapy 43. This concept of finding optimal administered doses was already applied for some radiopharmaceuticals 34-38, where PBPK predictions of different administered peptide amounts gave insights in the effects of these amounts on healthy tissue uptake and tumor exposure. However, in our view, its use can be expanded, since PBPK models are not regularly used to determine administered dosing regimens. This is a suitable alternative for time-consuming dose finding (i.e. injected peptide and activity) and organ distribution studies in patients and may lead to an evidence-based decision for the peptide amounts that will be used.

#### Radiopharmaceutical comparisons

Also for radiopharmaceutical selection, PBPK modelling is a useful tool for comparing PK of multiple compounds, as was discussed in one included article 52. This approach could also be of great interest for radiolabeled peptides, e.g. SSTR targeting peptides for NETs or PSMA targeted peptides for prostate cancer 66, 67. In both cases, there are many comparable radiopharmaceuticals with slight differences in physicochemical properties, resulting in different whole-body distribution profiles. PBPK modelling could help to predict those PK differences, but also provide a better understanding of why PK of these radiopharmaceuticals may differ, based on their chemical characteristics and e.g. affinity profiles. Obtaining insights in these aspects could then help to select preferable ligands specific for diseases or disease states.

#### Subgroup analysis

An unexplored feature of PBPK modelling is the extrapolation to special subgroups within a population. Using physiology information specific for a (patient) subgroup, PBPK models can be extrapolated to patient subgroups such as impaired elderly, pediatrics or patient with renal failure 11. This might eventually lead to adjusted administration protocols because of dissimilarities in radiopharmaceutical distribution.

#### (Dis)similarities with kinetic PET modelling

Regarding its application, PBPK modelling is quite similar compared to traditional kinetic PET modelling; both should help to describe PK of the applied radiopharmaceutical in specific compartments by defining the relationship between observed data and physiological parameters 68. Non-compartmental approaches are occasionally used for kinetic PET modelling, but most commonly a compartmental modelling approach is performed. However, compared to PBPK modelling, kinetic PET modelling applies concentration-time profiles in tissues of interest as input to develop a model ('top-down' approach) 68-70. Using these data, transport and binding rates of the radiopharmaceutical will be estimated by local concentration differences. Often, simplified compartmental tissue models are used, where the region(s) of interest are explicitly modelled. These compartmental models mostly contain 2-tissue (in case of transport markers) or 3-tissue compartments (in case of radiopharmaceuticals which are transported and undergo a metabolic step such as receptor or tissue binding) 70. However, blood will actually not represent a compartment, since blood input concentrations are treated as known values rather than predicted concentration values 68, 69. Linear regression is regularly used to mathematically solve those compartmental kinetic PET models, although in some (nonlinear) cases other techniques of numerical integration of differential equations are required 68, 70.

The main difference between this approach and PBPK models, next to the 'top-down' vs 'bottom-up' approach, is its complexity. Whole-body PBPK models are multi-compartment models and thus describe whole-body distribution, but also take into account many more parameters that have a physiological meaning. Therefore, PBPK models result in concentration-time profile predictions in the region of interest (where data were obtained), but predict these profiles for all other compartments as well using informative physiological parameters for all compartments specifically.

Compared to population PK modelling, which also is based on a 'top-down' approach, kinetic PET modelling approaches are mainly more simplistic and mathematically less complex. In most cases, general linear models are used in kinetic PET modelling compared to the use of NLMEMs in population PK modelling (see Supplementary Materials for additional information regarding NLMEMs). In other words, population PK modelling takes into account data of the population of interest by modelling data of all individuals simultaneously, but also, by including both fixed and random effects, variability on population parameters that are estimated. In addition, variability sources can be identified by covariate modelling. Also, in case of NLMEMs, input information is the administered dose (or activity) and thus the central compartment is explicitly modelled. This results in the advantage that CL predictions from this central compartment and covariates describing inter-patient variability on this CL can be described. To conclude, these advantages of using NLMEMs could be the answer to issues regarding the need to use population-based modelling (to automatically calculate input functions), as was addressed by Dimitrakopoulou-Strauss *et al.* 70.

#### Challenges

Drawbacks of PBPK modelling are mainly related to its model complexity. Many input parameters are required and the availability of such *in vitro* or pre-clinical data is limited. Besides, these parameters could differ between *in vitro* settings or non-human species and human species. Therefore, in most cases data is needed to evaluate, but also improve, model predictions. Nevertheless, assumptions still have to be performed and thus these must be clearly defined. Lastly, since these models represent the underlying physiology of an organism, it is essential to have a good understanding of this physiology and parameter optimization should be performed with caution and delineated within physiological logical boundaries.

## Conclusion

This review provided an overview of PBPK and population PK modelling of radiopharmaceuticals. Since publications of these modelling approaches are scarce in this field, the use of these approaches can be expanded to obtain a better understanding of PK and whole-body distribution of radiopharmaceuticals in general. These PK data are essential for eventually optimizing efficacy and safety of radiopharmaceutical therapies. In addition, PK modelling showed to be useful in answering more specific research questions, namely regarding treatment (planning) optimization, selection of ligand and ligand amounts, understanding drug-drug interactions, obtaining PK parameters and its variability within a population, and covariate modelling to identify factors or patient characteristics that contribute to significant differences in PK. In general, these studies will contribute to optimizing therapy of radiopharmaceuticals that are or will be used in clinical practice, mainly by leading to a more personalized or precision medicine approach. Other potential applications could be extrapolations to different patient subgroups, population dosimetry models, extrapolation of preclinical species to humans and optimizing risk-benefit ratios. To conclude, PK modelling of radiopharmaceuticals has great potential for the nearby future and could contribute to the evolving research of radiopharmaceuticals.

## Supplementary Material

Supplementary information and figure.

## Figures and Tables

**Table 1:** Results for population pharmacokinetic model publications regarding radiopharmaceuticals

Authors [reference]	Article title	Journal (year)	Radiopharmaceutical(s) for indication	Research purpose	Patients (n)	Data input (and source(s))	Model
Van Rij CM, Huitema AD, Swart EL, et al. 28	Population plasma pharmacokinetics of ^11^C-flumazenil at tracer concentrations	Br J Clin Pharmacol (2005)	[^11^C]C-flumazenil for localization of epileptic foci in patients with epilepsy and depression who are candidates for surgery	To develop a population PK model at tracer concentrations, to identify patient characteristics that influence its PK and to define and validate an optimal sampling protocol for [^11^C]C-flumazenil PET studies	51	Blood activity data (blood samples)	2-compartment model
Merrill S, Horowitz J, Traino AC, et al. 29	Accuracy and optimal timing of activity measurements in estimating the absorbed dose of radioiodine in the treatment of Graves' disease	Phys Med Biol (2011)	Radioiodine (^131^I) for Graves' disease	To study the accuracy of estimates of four target variables (time-integrated activity coefficient, time of maximum activity, maximum activity, and effective half-life in the gland) obtained with different sampling schedules	41	Activity uptake measurements (thyroid scan)	2-compartment model
Topić Vučenović V, Rajkovača Z, Jelić D, et al. 27	Investigation of influence of anti-thyroid drug discontinuation time on ^131^I biokinetics in patients with benign thyroid disease	Eur J Clin Pharmacol (2018)	Radioiodine (^131^I) for benign thyroid disease	To characterize biokinetics in patients with benign thyroid disease and to investigate and quantify the influence of patients' demographic and clinical characteristics on intra-thyroidal kinetics	345	Fractional thyroid gland uptake (thyroid uptake system)	2-compartment model
Puszkiel A, Bauriaud-Mallet M, Bourgeois R, et al. 25	Evaluation of the interaction of amino acid infusion on ^177^Lu-DOTATATE pharmacokinetics in patients with gastroenteropancreatic neuroendocrine tumors	Clin Pharmacokinet (2019)	[^177^Lu]Lu-DOTATATE for gastroenteropancreatic NETs	To develop a population PK model to analyze concentration-time data during and after amino acid (AA) co-infusion, to quantify the interaction of AA on [^177^Lu]Lu-DOTATATE PK and to evaluate the impact of plasma exposure on hematologic and renal toxicity	42	Blood activity data (blood samples)	3-compartment model
Melgar Pérez J, Orellana Salas A, Santaella Guardiola Y, et al. 30	Improving individualised dosimetry in radioiodine therapy for hyperthyroidism using population biokinetic modelling	Phys Med (2019)	Radioiodine (^123^I) for Graves' disease	To design and validate a population model applied to radioiodine biokinetics	58	Activity uptake measurements (dual gamma camera)	1-comparment model
Devasia TP, Dewaraja YK, Frey KA, et al. 31	A novel time-activity information-sharing approach using nonlinear mixed models for patient-specific dosimetry with reduced imaging time points: application in SPECT/CT after ^177^Lu-DOTATATE	J Nucl Med (2021)	[^177^Lu]Lu-DOTATATE for NETs	To compare a novel method for joint kidney time-activity estimation with previously proposed single-time-point methods in virtual and clinical patient data	10	Kidney activity data (SPECT/CT scans)	1-compartment model
Taprogge J, Carnegie-Peake L, Murray I, et al. 24	Adjustment of the iodine ICRP population pharmacokinetic model for the use in thyroid cancer patients after thyroidectomy	J Radiol Prot (2021)	Radioiodine ([^131^I]I-NaI) for thyroid cancer patients following thyroidectomy	To describe the PK of radioiodine in a thyroid cancer patient cohort based on actual patient data	23	Thyroid remnant, whole-body and blood activity retention data (SPECT scans and blood samples)	Multi-compartment model
Lambert M, Dierickx L, Brillouet S, et al. 26	Comparison of two types of amino acid solutions on ^177^Lu-DOTATATE pharmacokinetics and pharmacodynamics in patients with metastatic gastroenteropancreatic neuroendocrine tumors	Curr Radiopharm (2022)	[^177^Lu]Lu-DOTATATE for gastroenteropancreatic NETs	To compare the effect of two types of amino acid co-infusion on PK and toxicity of [^177^Lu]Lu-DOTATATE, and to determine the inter- and intra-individual variability	83	Plasma activity data (blood samples)	3-compartment model

Abbreviations: ICRP: International Commission on Radiological Protection; NET: neuroendocrine tumor; PK: pharmacokinetic. Publications are displayed in chronological publication date order.

**Table 2:** Results for physiologically based pharmacokinetic model publications regarding radiopharmaceuticals

Authors [reference]	Article title	Journal (year)	Radiopharmaceutical(s) (simulated) for indication	Research purpose	Patients used for model validation or simulations (virtual patients) (n)
Kletting P, Bunjes D, Reske SN, et al. 37	Improving anti-CD45 antibody radioimmunotherapy using a physiologically based pharmacokinetic model	J Nucl Med (2009)	Anti-CD45 antibody YAML568 for acute myeloid leukemia	To individually determine the optimal preload for radioimmunotherapy to find the most favorable biodistribution for each patient	5
Kletting P, Muller B, Erentok B, et al. 43	Differences in predicted and actually absorbed doses in peptide receptor radionuclide therapy	Med Phys. (2012)	[^90^Y]Y-DOTATATE for NETs	To investigate the conjectured differences between pre-therapeutic and therapeutic biodistribution	10
Pfeifer ND, Goss SL, Swift B, et al. 51	Effect of ritonavir on ^99m^Tc-mebrofenin disposition in humans: A semi-PBPK modelling and *in vitro* approach to predict transporter-mediated DDIs	CPT Pharmacometrics Syst Pharmacol (2013)	[^99m^Tc]Tc-mebrofenin for diagnosis of structural and functional disorders of the hepatobiliary network and gallbladder	To describe a unique blood, liver, and bile clinical data set and to simulate sites/mechanisms of a [^99m^Tc]Tc-mebrofenin-ritonavir drug-drug interaction	18
Kletting P, Maaß C, Reske S, et al. 46	Physiologically based pharmacokinetic modelling is essential in ^90^Y-labeled anti-CD66 radioimmunotherapy	PLoS ONE (2015)	^90^Y-labelled anti CD66 antibodies for acute leukemia	To demonstrate: 1) the need to perform patient-specific dosimetry in ^90^Y-labeled anti-CD66 radioimmunotherapy, 2) that pre-therapeutic and therapeutic biodistributions differ, 3) that this difference in biodistributions can be accurately predicted	27
Hardiansyah D, Maass C, Attarwala AA, et al. 47	The role of patient-based treatment planning in peptide receptor radionuclide therapy	Eur J Nucl Med Mol Imaging (2016)	[^90^Y]Y-DOTATOC for metastasized NETs	To investigate the role of patient-based treatment planning in peptide receptor radionuclide therapy	15
Kletting P, Kull T, Maaß C, et al. 38	Optimized peptide amount and activity for ^90^Y-labeled DOTATATE therapy	J Nucl Med (2016)	[^90^Y]Y-DOTATATE for metastasizing NETs or meningioma	To develop a treatment planning approach that allows for the estimation of the optimal combination of amount and activity for peptide receptor radionuclide therapy for a given maximal kidney biologically effective dose	9
Gospavic R, Knoll P, Mirzaei S, et al. 33	Physiologically based pharmacokinetic (PBPK) model for biodistribution of radiolabeled peptides in patients with neuroendocrine tumours	Asia Oceania J Nucl Med Biol (2016)	[^177^Lu]Lu-DOTATATE for NETs	To assess the benefits of the application of PBPK models in patients with different neuroendocrine tumors	4
Hardiansyah D, Begum NJ, Kletting P, et al. 41	Sensitivity analysis of a physiologically based pharmacokinetic model used for treatment planning in peptide receptor radionuclide therapy	Cancer Biother Radiopharm. (2016)	[^111^In]In-DTPAOC for metastasized NETs	To evaluate the sensitivity of the time-integrated activity coefficients values on the erroneously chosen fixed parameters	15
Hardiansyah D, Guo W, Kletting P, et al. 42	Time-integrated activity coefficient estimation for radionuclide therapy using PET and a pharmacokinetic model: A simulation study on the effect of sampling schedule and noise	Med Phys (2016)	[^90^Y]Y-DOTATATE for metastasized NETs	To investigate the feasibility to predict time-integrated activity coefficients during peptide receptor radionuclide therapy based on PET data	15
Maass C, Sachs JP, Hardiansyah D, et al. 45	Dependence of treatment planning accuracy in peptide receptor radionuclide therapy on the sampling schedule	EJNMMI Res (2016)	[^111^In]In-DTPAOC for NETs	To investigate the effect of reduced number of measurement points on treatment planning accuracy in peptide receptor radionuclide therapy	15
Hardiansyah D, Attarwala AA, Kletting P, et al. 40	Prediction of time-integrated activity coefficients in PRRT using simulated dynamic PET and a pharmacokinetic model	Phys Med (2017)	[^90^Y]Y-DOTATATE for metastasized NETs	To investigate the accuracy of predicted time-integrated activity coefficients in peptide receptor radionuclide therapy using simulated dynamic PET data and PBPK model	15
Begum NJ, Thieme A, Eberhardt N, et al. 35	The effect of total tumor volume on the biologically effective dose to tumor and kidneys for ^177^Lu-labeled PSMA peptides	J Nucl Med (2018)	[^177^Lu]Lu-PSMA I&T for metastatic castration-resistant PCa	To quantitatively investigate the effects of different total tumor volumes and varying activities and peptide amounts on the biologically effective doses to tumors and organs at risk	13
Jiménez-Franco LD, Kletting P, Beer AJ, et al. 36	Treatment planning algorithm for peptide receptor radionuclide therapy considering multiple tumor lesions and organs at risk	Med Phys (2018)	[^177^Lu]Lu-DOTATATE for metastasizing NETs or meningioma	To develop a clinically applicable algorithm for treatment planning in peptide receptor radionuclide therapy	9 (virtual patients)
Kletting P, Thieme A, Eberhardt N, et al. 48	Modelling and predicting tumor response in radioligand therapy	J Nucl Med (2019)	[^177^Lu]Lu-PSMA I&T for metastatic castration-resistant PCa	To develop a theranostic method that allows predicting tumor volume after radioligand therapy	13
Rinscheid A, Lee J, Kletting P, et al. 44	A simulation-based method to determine optimal sampling schedules for dosimetry in radioligand therapy	Z Med Phys (2019)	[^111^In]In-DOTATATE for metastasising meningioma and metastasising NETs	To develop a general and flexible method, which analyses numerous clinically applicable sampling schedules	9 (virtual patients)
Begum NJ, Glatting G, Wester HJ, et al. 34	The effect of ligand amount, affinity and internalization on PSMA-targeted imaging and therapy: A simulation study using a PBPK model	Sci Rep (2019)	^68^Ga-labelled and ^177^Lu-labelled PSMA-specific ligands for metastatic castration-resistant PCa	To investigate the interconnected effect of affinity, internalization and injected ligand amount of PSMA-specific ligands	13 (virtual patients)
Jiménez-Franco LD, Glatting G, Prasad V, et al. 50	Effect of tumor perfusion and receptor density on tumor control probability in ^177^Lu-DOTATATE therapy: An in silico analysis for standard and optimized treatment	J Nucl Med (2021)	[^177^Lu]Lu-DOTATATE for NETs and meningioma	To determine minimal tumor perfusion and receptor density considering a desired tumor control probability of 99% and biologically effective doses for organs at risk	9 (virtual patients)
Hardiansyah D, Kletting P, Begum NJ, et al. 49	Important pharmacokinetic parameters for individualization of ^177^Lu-PSMA therapy: A global sensitivity analysis for a physiologically-based pharmacokinetic model	Med Phys (2021)	^177^Lu-labelled PSMA-targeting ligands for metastatic castration-resistant PCa	To identify influential anatomical and/or physiological parameters that have a large contribution to the inter-individual differences of the absorbed doses in kidney and tumor lesions	13
Siebinga H, De Wit-van der Veen BJ, Beijnen JH, et al. 32	A physiologically based pharmacokinetic (PBPK) model to describe organ distribution of ^68^Ga‑DOTATATE in patients without neuroendocrine tumors	EJNMMI Res (2021)	[^68^Ga]Ga-DOTATATE for patients without detectable NETs	To describe organ distribution of [^68^Ga]Ga-DOTATATE in a population of patients without detectable NETs	41
Bartelink IH, Van de Stadt EA, Leeuwerik AF, et al. 52	Physiologically based pharmacokinetic (PBPK) modeling to predict PET image quality of three generations EGFR TKI in advanced-stage NSCLC patients	Pharmaceuticals (2022)	[^11^C]C-erlotinib, [^18^F]F-afatinib and [^11^C]C-osimertinib	To predict the image quality by predicting the right tumor-to-lung contrast and to predict the whole-body distribution	19

Abbreviations: DDI: drug-drug interaction; NET: neuroendocrine tumor; NSCLC: non-small cell lung cancer; PBPK model: physiologically based pharmacokinetic model; PCa: prostate cancer; PRRT: peptide receptor radionuclide therapy; PSMA: prostate specific membrane antigen. Publications are displayed in chronological publication date order.

## Abbreviations

^11^C: Carbon-11
^111^In: Indium-111
^123^I: Iodine-123
^131^I: Iodine-131
^177^Lu: Lutetium-177
^18^F: Fluorine-18
^68^Ga: Gallium-68
^90^Y: Yttrium-90
^99m^Tc: Technetium-99m
BED: biological effective dose
CL: clearance
CPI: critical path initiative
DDI: drug-drug interaction
EANM: european association of nuclear medicine
EGFR: epidermal growth factor receptor
EMA: european medicines agency
FDA: food and drug administration
ICRP: international commission on radiological protection
IVIVE: *in vitro-in vivo* extrapolation
K_D_: dissociation constant
k_off_: dissociation rate constant
k_on_: association rate constant
MIDD: model-informed drug development
NET: neuroendocrine tumor
NLMEM: nonlinear mixed-effects model
OAR: organ at risk
PBPK: physiologically based pharmacokinetic
PD: pharmacodynamic(s)
PK: pharmacokinetic(s)
PRRT: peptide receptor radionuclide therapy
PSMA: prostate-specific membrane antigen
RDI: radiopharmaceutical development initiative
SSTR: somatostatin receptor
TIAC: time-integrated activity coefficient
TKI: tyrosine kinase inhibitor
