# Supplementary material for: Current use and future potential of (physiologically based) pharmacokinetic modelling of radiopharmaceuticals: a review
Source: Theranostics. 2022 Nov 14;12(18):7804–20. doi: 10.7150/thno.77279 (PMC9706588; doi:10.7150/thno.77279)
Supplement: Supplementary file 1 — Supplementary information and figure. [file thnov12p7804s1.pdf]

Supplementary Materials

Current use and future potential of (physiologically based) pharmacokinetic modelling of radiopharmaceuticals: a review

Hinke Siebinga[1,2], Berlinda J. de Wit-van der Veen[2], Marcel D.P. Stokkel[2], Alwin D.R. Huitema[1,3,4], Jeroen J.M.A. Hendrikx[1,2]

1 Department of Pharmacy & Pharmacology, The Netherlands Cancer Institute, Amsterdam, The Netherlands

2 Department of Nuclear Medicine, The Netherlands Cancer Institute, Amsterdam, The Netherlands

3 Department of Clinical Pharmacy, University Medical Center Utrecht, Utrecht University, Utrecht, The Netherlands

4 Department of Pharmacology, Princess Máxima Center for Pediatric Oncology, Utrecht, The Netherlands

Corresponding author: j.hendrikx@nki.nl, Plesmanlaan 121, 1066 CX Amsterdam, tel +31205124481

Literature search terms

Detailed information on the search terms for both population PK and PBPK modelling is provided below. For PubMed search terms, '[tiab]' refers to title and abstract. For Embase search terms, 'ti,ab,kw' refers to title, abstract and keywords.

Population PK modelling

PubMed:

("population pharmacokinetic*"[tiab] OR PopPK[tiab] OR "pop pk"[tiab] OR NONMEM[tiab] OR "nonlinear mixed effects model*"[tiab]) AND ("Radiopharmaceuticals"[Pharmacological Action] OR "Radiopharmaceuticals"[Mesh] OR "Radioactive Tracers"[Mesh] OR "Radioactivity"[Mesh] OR radiopharmaceutical*[tiab] OR tracer*[tiab] OR radioactiv*[tiab])

Embase:

('population pharmacokinetics'/exp OR 'pharmacokinetic modelling software'/exp OR ("population pharmacokinetic*" OR PopPK OR NONMEM OR "nonlinear mixed effects model*"):ti,ab,kw) AND ('radiopharmaceutical agent'/exp OR 'radioactivity'/exp OR (radiopharmaceutical* OR tracer* OR radioactiv*):ti,ab,kw)

PBPK modelling

PubMed:

("physiologically based pharmacokinetic*"[tiab] OR PBPK[tiab]) AND ("Radiopharmaceuticals"[Pharmacological Action] OR "Radiopharmaceuticals"[Mesh] OR "Radioactive Tracers"[Mesh] OR "Radioactivity"[Mesh] OR radiopharmaceutical*[tiab] OR tracer*[tiab] OR radioactiv*[tiab])

Embase:

('physiologically based pharmacokinetic model'/exp OR ("physiologically based pharmacokinetic*" OR PBPK):ti,ab,kw) AND ('radiopharmaceutical agent'/exp OR 'radioactivity'/exp OR (radiopharmaceutical* OR tracer* OR radioactiv*):ti,ab,kw)

Additional information

Population PK modelling

Population PK studies are most frequently performed by nonlinear mixed-effects models (NLMEMs). Nonlinear means that the concentration, as a dependent variable, is nonlinearly related to the (estimated) parameters and independent variable(s). Mixed-effects refer to taking both fixed and random effects into account. Fixed effects do not vary over time and represent typical population PK parameter values, such as clearance and volume of distribution [1]. In addition, individual characteristics that cause the typical PK parameters to vary across the population are also fixed effects, because the effect of a characteristic on a parameters is estimated for the whole population. On the other hand, random effects lead to differences between expectations and outcomes in individual patients [2]. Random effects include inter-individual variability (between subject variability (BSV)), between occasion variability (BOV) and residual variability [1,3]. Residual variability can be categorized as a combination of variability, such as intra-individual variability, drug concentration measurement error, sampling time error and model misspecification error [2]. NLMEMs use the population, rather than the individual, as the unit of analysis for estimating PK parameters and random effects [4,5]. In other words, data from all individuals are modelled simultaneously instead of separately.

These population PK models contain three main components: a structural model, a stochastic (or statistical) model and covariate models. Herewith the typical concentration-time course, the random effects in concentration and the variability predicted by participant characteristics (covariates) are described, respectively [1,3]. NLMEMs result in typical population parameter estimates (with optional inter-patient variability on specific parameters) and a residual variability. Using these estimates, population concentration-time profiles are estimated, but also individual predictions of PK parameters and concentration-time profiles based on this population prediction can be provided. An overview of the NLMEM structure and fixed and random effect is provided in Figure S1.

There are multiple ways to evaluate NLMEMs, but regarding simplicity, only three main but simple evaluation methods will be briefly described here. Firstly, the objective function value (OFV) is

used to discriminate between models during early stages of model development. The OFV, minus twice the log of the likelihood, is a value that gives an estimate of how good the model predictions describe the data. However, the OFV depends on the data set and the estimation method that is used [1]. Furthermore, goodness-of-fit plots are used for visual model evaluation. These plots show, for example, (conditional weighted) residuals versus population predicted concentrations and observed versus population or individual predicted concentrations. Besides, a visual predictive check (VPC) is a simulation-based method to evaluate models. Prediction intervals are developed from simulation concentration-time profiles and these are then compared with observed data [1].

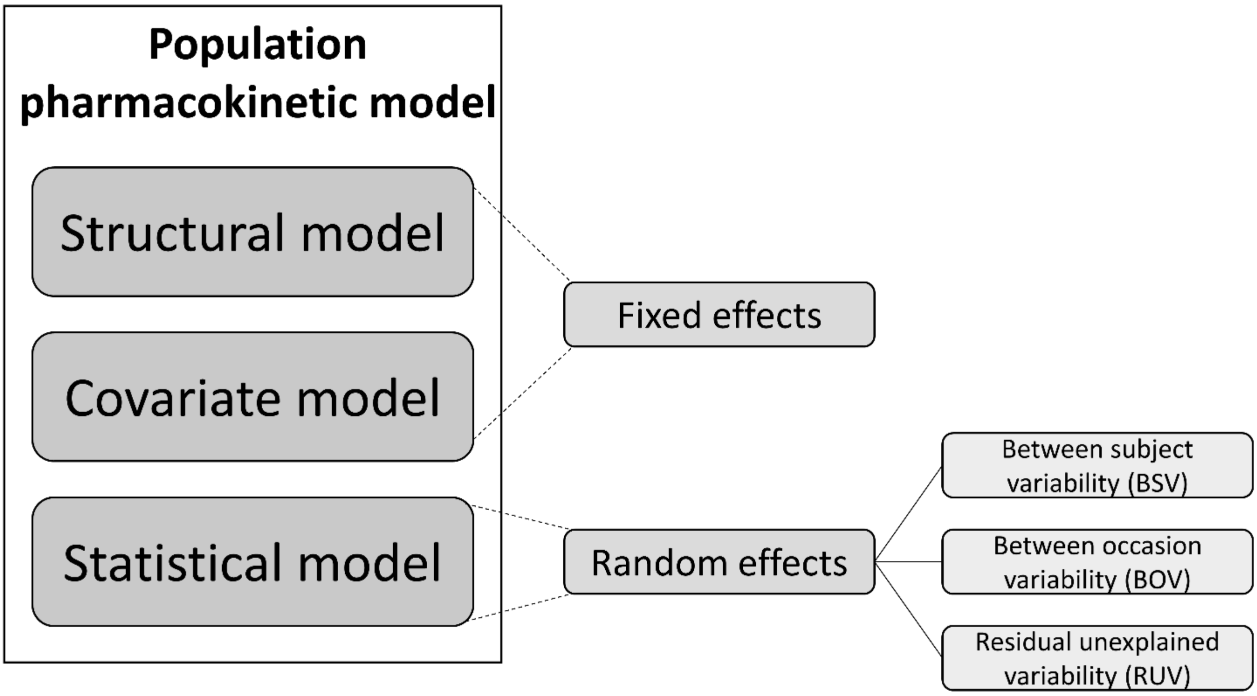

Figure S1 – Overview of nonlinear mixed-effects model (NLMEM) structure and how fixed and random effects are involved.

References

1. Mould DR, Upton RN. Basic concepts in population modeling, simulation, and model-based drug development-part 2: introduction to pharmacokinetic modeling methods. CPT Pharmacometrics Syst Pharmacol. 2013; 2: e38.

2. Ette EI, Williams PJ. Population pharmacokinetics I: background, concepts, and models. Ann Pharmacother. 2004; 38: 1702-6.

3. Mould DR, Upton RN. Basic concepts in population modeling, simulation, and model-based drug development. CPT Pharmacometrics Syst Pharmacol. 2012; 1: e6.

4. Kiang TKL, Sherwin CMT, Spigarelli MG, Ensom MHH. Fundamentals of population pharmacokinetic modelling. Clin Pharmacokin. 2012; 51: 515-25.

5. Roy A, Ette EI. A pragmatic approach to the design of population pharmacokinetic studies. AAPS J. 2005; 7: E408-20.
